# The self-efficacy in distress tolerance scale (SE-DT): a psychometric evaluation

**DOI:** 10.1186/s40479-022-00195-9

**Published:** 2022-10-10

**Authors:** Sven Alfonsson, Karolina Mardula, Christine Toll, Martina Isaksson, Martina Wolf-Arehult

**Affiliations:** 1 Department of Clinical Neuroscience, Centre for Psychiatry Research, Karolinska Institutet and Stockholm City County, SE-171 77 Stockholm, Sweden; 2 Psychiatry Northwest, PO Box 98, Region Stockholm, SE-191 22 Sollentuna, Sweden; 3 Department of Neuroscience, Psychiatry, Uppsala University, Entrance 10, Floor 3B, SE-751 85 Uppsala, Sweden

**Keywords:** DBT skills training, Distress tolerance, Emotional dysregulation, Self-efficacy, Psychometric evaluation, Borderline personality disorder

## Abstract

**Background:**

Skills training is believed to be essential in dialectical behavior therapy (DBT) and is also offered as a standalone intervention. There is a need to better understand each skills module’s separate contribution to treatment outcomes. Several assessment instruments are available, but none of them provides specific information about patients’ perceived ability to use skills promoting distress tolerance. The aim of the present study was to develop and evaluate the psychometric properties of a Swedish adaptation of the General Self-Efficacy scale (GSE) for skills use in distress tolerance – the Self-Efficacy in Distress Tolerance scale (SE-DT).

**Methods:**

Cross-sectional and longitudinal data were gathered in a non-clinical (NC) community sample (*n* = 407) and a clinical psychiatric (CP) sample (*n* = 46). Participants in the NC sample were asked to complete a set of 19 self-report instruments, including the SE-DT, and 45 participants repeated the assessment after 2 weeks. The patients in the CP sample filled out a subset of eight instruments; twenty patients repeated the assessment after completing a treatment intervention including mindfulness skills and distress tolerance skills or emotion regulation skills.

**Results:**

The analyses showed that the SE-DT is unidimensional with high internal consistency (Cronbach’s alpha = .92) and good test-retest reliability (intraclass correlation = .74). The SE-DT also showed good convergent and divergent validity, demonstrating positive correlations with general self-efficacy and self-compassion, and negative correlations with difficulties in emotion regulation, psychiatric symptoms, and borderline symptoms. The SE-DT showed sensitivity to change, when pre- and post-treatment assessments were compared (Cohen’s *d* = 0.82).

**Discussion:**

This is preliminary evidence that the SE-DT has adequate to good psychometric properties, supporting the use of a total sum score. The results indicate that the SE-DT can adequately measure the construct of self-efficacy with regard to dealing with distress and emotional crises. The instrument enables continued investigation of standalone skills training and the specific contribution of distress tolerance skills to treatment outcomes in DBT. Further studies are needed to investigate whether these results are valid in other populations. In addition, the field would benefit from a common definition of distress tolerance.

## Introduction

Difficulties in emotion regulation are considered a core feature of borderline personality disorder (BPD), triggering dysfunctional behaviors such as suicide attempts and self-harm [26, 32]. Dialectical behavior therapy (DBT) is a comprehensive treatment that targets these behaviors, helping BPD patients increase behavioral control and build a life worth living [32]. In addition to receiving individual therapy and telephone coaching, patients participate in a skills training group where they learn a wide range of skills grouped into four modules: emotion regulation, mindfulness, interpersonal effectiveness, and distress tolerance (DT) [31]. In recent years, the use of skills has been suggested as a key mechanism of change in DBT; for example, more frequent use of skills is associated with less frequent self-harm and dropout [6, 38, 51]. For some BPD patients, a standalone skills training group may be an effective intervention [37]. This may be the case in particular if higher risk patients are excluded [35]. Further, it has been reported that a standalone skills training group can be implemented across several different clinical settings and populations [54]. In some cases, individual online skills training may be a convenient treatment format in preparation and while waiting for standard DBT (i.e., DBT programs that include all treatment modes) (Vasiljevic S, Isaksson M, Wolf-Arehult M, Oster C, Ramklint M, Isaksson J. Brief internet-delivered skills training based on DBT for adults with borderline personality disorder – a qualitative study. Submitted). However, additional research on standalone skills training is needed, with improved research methodology; for example, it is important to improve the assessment of skills [54]. Valid and reliable assessment instruments are also a prerequisite for investigating if and how different skills modules are associated with treatment outcome and dropout in standard DBT. Although DBT is clearly effective as a treatment for BPD, only about 40% of patients are clinically recovered on general measures of psychopathology [9] and dropout rates are about 20% [33]. Thus, there is a need to improve the treatment.

Currently, there are several well-known and evaluated instruments available for the assessment of DBT skills. Linehan and colleagues developed the Dialectical Behavior Therapy Ways of Coping Checklist (DBT-WCCL), assessing coping via DBT skills, as well as dysfunctional behaviors [39]. However, this instrument involves only general assessment of skills use and is not able to differentiate between skills from different modules. In addition, emotion regulation skills have often been assessed with the Difficulties in Emotion Regulation Scale (DERS [8, 19];), interpersonal skills with the Inventory of Interpersonal Problems (IIP [30];), and DT skills with the Distress Tolerance Scale (DTS [50];). However, these questionnaires assess self-perceived problems, rather than the use of effective DBT skills. The following items can exemplify this: “When I’m upset, I have difficulty focusing on other things” (DERS) and “My feelings of distress or being upset scare me” (DTS). In contrast, mindfulness skills have been assessed with instruments such as the Kentucky Inventory of Mindfulness Skills (KIMS [2, 44];), demonstrating a more direct way of investigating the use of mindfulness skills: “When I’m doing something, I’m only focused on what I’m doing, nothing else” (KIMS). Thus, there is a need to develop additional instruments focusing on the assessment of skills use related to different skills.

The skills from the DT module may be the most difficult to assess, since there is no conclusive consensus on how to conceptualize or assess DT [28]. It has been suggested that DT represents an individual’s ability to handle uncomfortable emotion states and sensations [53]. In DBT, DT is defined in a more precise way as the ability to accept (tolerate) distress when it is unavoidable and to handle emotional crises in an effective way [31]. BPD pathology has been reported to be associated with low DT and difficulties in emotion regulation [21]. However, it has also been reported that BPD patients who exhibit high DT are at greater risk of engaging in serious suicidal behaviors, i.e., in some cases it is possible that a strong ability to tolerate distress enables the patient to perform otherwise unsustainable behaviors [1]. In other words, it seems that DT can sometimes be functional, and at other times dysfunctional. In particular, DT strategies become dysfunctional when they are performed instead of effective emotion regulation and problem solving (the failure to use emotion regulation and problem solving may very well be the main cause of distress in the first place). Interestingly, it has been suggested that DT lies at the intersection of emotion and self-control, and that lower willpower self-efficacy predicts subsequent distress intolerance [56]. It has also been suggested that DT is a trait-like construct, not likely to change over time [24].

A problem when assessing DBT skills is that patients are not familiar with these skills prior to treatment; asking patients about something they do not yet understand can make pre-treatment assessments difficult. In addition, it is difficult to compare patients receiving treatment-as-usual with patients receiving DBT if we investigate something that patients learn about in DBT (e.g., DBT skills). Therefore, researchers have underlined the importance of not using DBT-specific terminology in assessment instruments [6, 38]. Another solution to this problem is to ask patients indirectly about their ability to use skills, that is, to assess their perceived self-efficacy (SE).

SE refers to an individual’s belief in their ability to perform a specific behavior when confronted with a certain problem, and rests on the premise that an individual only engages in a behavior if they believe in their own ability to perform the behavior and in the possibility that the behavior leads to a desired goal [3]. During psychotherapy, SE may determine what challenges the patient takes on and how much they strive to achieve a goal [5]. In general, SE has been associated with positive affect and high life satisfaction [27, 34], and has been described as having a predictive role in health-promoting behaviors [49]. According to Bandura [3], SE differs between different areas of life, i.e., one and the same person can report a high level of SE in one area, but a low level of SE in another. Unsurprisingly, high SE in one area is believed to be generalizable to similar areas, as specific underlying skills can be relevant to several areas [3]. It has been suggested that SE consists of a general inherent belief in one’s own ability that is expressed within a broad spectrum of life’s various domains, rather than a belief in one’s ability within a defined area [55]. However, a domain-specific assessment of SE may be a stronger predictor of specific behaviors than an assessment of general SE [36]. A change in domain-specific SE during psychotherapy might also be easier to detect than changes in general SE. Indeed, our clinical experience shows that patients may report different SE levels in different areas. For example, a patient may report a high level of SE when it comes to supporting other people in distress and suggesting skills to use in such situations, while reporting major difficulties in applying the same expertise to themselves, i.e., reporting a low level of SE regarding DT skills in their own life. Patients may also report a high level of SE in working life, even though their private life is characterized by a low level of SE, as well as repeated self-harm and suicidal behaviors.

Since DBT includes skills training in a group format, it offers an excellent opportunity to improve SE levels. The reason is that SE is enhanced in group settings where patients see other patients master difficulties similar to those they are experiencing themselves [4]. SE is also associated with positive psychotherapeutic outcome in other psychiatric disorders, including substance use [22], social phobia [18], and anxiety disorders [13]. Skills use and SE have independently been associated with less frequent self-harm in BPD patients [6].

A commonly used measure of general SE is the General Self-Efficacy scale (GSE [47];) which is a 10-item self-report questionnaire with good to excellent psychometric properties that has been used in many studies and countries [34]. Previous studies indicate that the GSE is unidimensional with an average Cronbach’s alpha of .86 [45]. Evaluations of the Swedish version of the GSE have shown comparable results [14, 36]. The wording of the GSE items is possible to understand without having attended DBT skills training: *No matter what comes my way, I’m usually able to handle it.* The developers of the GSE recommend that the scale is adapted to the specific area of interest [46]. Importantly, studies conducted in several countries have shown that focusing on general SE, rather than specific SE, decreases the instrument’s sensitivity [34].

In summary, there is a need to develop assessment instruments focusing on the use of DBT skills from different skills modules. This will enable us to better understand each skills module’s separate contribution to treatment outcome and dropout in standalone skills training and skills training in standard DBT. Several instruments related to specific skills modules or the entire skills training are available, but to our knowledge none of them provides specific information about patients’ perceived ability to use skills to deal with distress and emotional crises. Assessment of SE in DT could therefore make an important contribution to future treatment studies. The aim of the present study was to develop and evaluate the psychometric properties of a Swedish adaptation of the GSE for skills use in DT – the Self-Efficacy in Distress Tolerance scale (SE-DT). We hypothesized that the SE-DT would show satisfactory to high internal consistency and test-retest reliability, and a factor structure indicating a single scale. Further, we hypothesized satisfactory convergent and divergent construct validity when the SE-DT was compared with other instruments, such as positive correlations with the GSE and self-compassion and negative correlations with psychiatric and BPD symptoms. The SE-DT was also hypothesized to show a significantly lower mean value in a clinical psychiatric (CP) sample than in a non-clinical (NC) community sample, and to be sensitive to treatment change in a CP sample after participation in a brief skills training program.

## Methods

### Procedure and participants

Data for psychometric analyses were collected in a NC community sample, using a set of 19 online self-report instruments, and a CP sample, using a subset of eight self-report instruments. Participants in the NC sample were recruited via convenience sampling using a website specifically designed for gathering participants for scientific research and advertisements on social media platforms. Interested participants signed up for more information and then received an e-mail with a link to more information about the study. Those who chose to participate were asked to sign an informed consent form and fill out the study instruments and background variables online. Two weeks after the first assessment, a subsample (*n* = 45) filled out the questionnaires a second time, for test-retest analysis. Participants in the NC sample were offered remuneration in the form of a movie ticket voucher for each assessment. All data were collected anonymously. In total, 407 people participated and filled out the instruments. The background variables are presented in Table 1.

**Table 1:** Background variables for the non-clinical community sample (*n* = 407)

*Gender*	N
Female	303 (75%)
Male	103 (25%)
Other	1 (0%)
*Age (years)*	*M* = 30.5 (range: 18–77; SD = 10.66)
*Marital status*	N
Single	164 (40%)
Married/cohabitant	173 (43%)
Partner but not living together	44 (11%)
Single with children	11 (3%)
Other	12 (3%)
*Education*	
Elementary	10 (3%)
High school/College	111 (27%)
University	251 (62%)
Other	35 (8%)
*Occupation*	
Working	166 (41%)
Studying	187 (46%)
Unemployed	15 (3%)
Sick leave	16 (4%)
Parental leave	3 (1%)
Other	20 (5%)

Participants in the CP sample were recruited at two sites: a general psychiatric outpatient clinic, primarily treating patients with depression, anxiety disorders, and personality disorders, and the BPD unit at Psychiatry Northwest in Stockholm, treating patients with full or sub-threshold (fulfilling four diagnostic criteria) BPD. During new patients’ initial appointment with a psychiatrist or a psychologist, health professionals routinely asked them to participate in diagnostic interviews, as well as to fill out a standard battery of self-report instruments. Diagnosis was based on the Mini-International Neuropsychiatric Interview (M.I.N.I.) [48] and the Structured Clinical Interview for DSM-IV Axis II Personality Disorders, borderline criteria (SCID-II) [17]. Patients were informed about the study during the diagnostic evaluation.

A total of 30 patients at the general psychiatric outpatient clinic, of whom 26 (87%) were female and 4 (13%) were male, chose to participate, signed an informed consent form, and completed the instruments in a baseline assessment. For 22 of these patients, the mean number of fulfilled BPD criteria was four; the remaining patients had depression or anxiety disorders. The patients with BPD-typical symptoms and/or self-harm were offered to participate in a standalone full-scale skills training group, including 24 sessions, or a standalone brief skills training group, including eight sessions (focusing only on mindfulness skills and DT skills), depending on in which group started next and the treatment goals of the patient. Twenty patients agreed to start treatment and filled out the questionnaires again in a second assessment after completing the skills group.

Sixteen patients at the BPD unit, thirteen female (81%) and three male (19%), chose to participate. The mean number of fulfilled BPD criteria was four. Seven of these patients participated in one of three treatment options: a standalone full-scale skills training group, including 24 sessions, a DBT program with both skills training and individual therapy (6–12 months), or emotion regulation group therapy (ERGT [43];) including 16 sessions. They filled out the questionnaires both before and after completing treatment. Standalone skills training and ERGT were offered to patients with less severe and subthreshold BPD, whereas the DBT program was offered to patients with more severe problems and self-harm.

In total, there were 46 patients in the CP sample and 27 of them provided data for the sensitivity analysis. The patients in the CP sample were between 18 and 54 years old, with a mean age of 28.15 years (SD = 7.8).

### The self-efficacy scale for distress tolerance

The *SE-DT* assesses a person’s perceived SE in dealing with distress and emotional crises, focusing on the use of effective skills and avoiding dysfunctional and impulsive behaviors with negative consequences (such as self-harm) or doing things one later regrets. The SE-DT is an adaptation of the GSE [47]. The adaptation was made by the fourth and last authors, changing or adding some words in the ten items to ensure that the scale would assess the specific domain in question (see Table 2). Like the original scale, the SE-DT consists of ten items rated on a 4-point Likert scale, ranging from 1 (“not at all true”) to 4 (“exactly true”). The scale is hypothesized to be unidimensional and generates a sum score, ranging from 10 to 40. A higher score indicates a higher level of perceived SE.

**Table 2:** The wording of the instruction and the items in the self-efficacy for distress tolerance scale (SE-DT), as well as the original items in the General Self-Efficacy scale (GSE)

Instruction SE-DT: *The statements below are about how to deal with emotional crises and whether you have effective strategies, that is, helpful strategies that do* *not* *lead to negative consequences. Please rate how well each item describes you.*	
SE-DT	GSE
I can always manage to *deal with emotional crises* if I try hard enough.	I can always manage to solve difficult problems if I try hard enough.
If someone opposes me *during an emotional crisis*, I can find the means and ways *to reach my goals.*	If someone opposes me, I can find the means and ways to get what I want.
It is easy for me to stick to my aims and accomplish my goals *despite emotional crises.*	It is easy for me to stick to my aims and accomplish my goals.
I am confident that I could deal efficiently with unexpected *crisis situations*.	I am confident that I could deal efficiently with unexpected events.
Thanks to my resourcefulness, I know how to handle unforeseen *crisis situations and without acting destructively or impulsively*.	Thanks to my resourcefulness, I know how to handle unforeseen situations.
I can *deal with* most *emotional crises* if I invest the necessary effort.	I can solve most problems if I invest the necessary effort.
I can remain calm when facing *emotional crises* because I can rely on my coping abilities.	I can remain calm when facing difficulties because I can rely on my coping abilities.
When I am confronted with *an emotional crisis*, I can usually find several solutions.	When I am confronted with a problem, I can usually find several solutions.
If I am in trouble *and it triggers strong negative emotions*, I can usually think of a solution.	If I am in trouble, I can usually think of a solution.
I can usually handle whatever comes my way *and without doing anything that I then regret.*	I can usually handle whatever comes my way.

### Instruments

The *Borderline Symptom List – short version (BSL-23)* is a self-report questionnaire targeting experiences and emotions that are typical of BPD patients [10]. In the BSL-23, patients are asked to assess 23 items with regard to the preceding week on a 5-point Likert scale, ranging from 0 (“not at all”) to 4 (“very strong”). A total mean score between 0 and 4 is used in evaluations, where a higher score indicates stronger BPD symptoms. A mean score of 2.05 (SD = 0.90) has been reported among BPD patients and the internal consistency for the instrument is high, with a Cronbach’s alpha of .94–.97 [10]. These results are comparable to those of the original scale (BSL-95 [11];). In addition, comparisons between pre- and post-treatment assessment scores showed improvements with a large effect size for the BSL-23 (*d* = 0.47) after 3 months of inpatient DBT [10]. These psychometric properties have been replicated in several studies, investigating different translations of the BSL-23 [25]. In the current study, the alpha value of the BSL-23 was .92.

The *Difficulties in Emotion Regulation Scale (DERS)* is a 36-item self-report questionnaire and was developed to measure difficulties with emotion regulation. The original version of the scale has shown good psychometric properties [19], as has the shortened 16-item version (DERS-16 [8];). Patients are asked to assess the items with regard to the preceding week on a 5-point Likert scale, ranging from 1 (“almost never”) to 5 (“almost all the time”). The DERS-16 generates a sum score that ranges from 16 to 80, where a higher score indicates more difficulties in emotion regulation. The DERS-16 has demonstrated excellent internal consistency with a Cronbach’s alpha of .92–.94 [8, 20]. In the current study, the alpha value of the DERS-16 was .95.

The *General Self-Efficacy scale (GSE)* was developed as a self-report instrument, using ten items to assess, on a 4-point Likert scale, a person’s general belief that they can perform a difficult task and cope with adversities in life [47]. Thus, the total score of the GSE ranges from 10 to 40; higher scores indicate a greater sense of general SE. The GSE is available in over 30 languages and the original Swedish version of the GSE was developed by Koskinen-Hagman, Schwarzer, and Jerusalem (http://userpage.fu-berlin.de/~health/selfscal.htm). Both the original version [45] and the Swedish version [14, 36] of the GSE have been reported to have good psychometric properties. In the current study, the alpha value of the GSE was .92.

The *Hopkins Symptom Checklist (HSCL-25)* includes 25 items and assesses both anxiety (10 items) and depression (15 items) during the preceding week on a 4-point Likert scale, ranging from 1 (“not at all”) to 4 (“a lot”). The scale generates a total mean score, ranging from 1 to 4, with higher scores indicating higher levels of anxiety and depression. It is a short version of the original version of the HSCL [15] and has shown adequate psychometric properties [16, 41]. In the current study, the alpha value of the HSCL-25 was .95.

The *Self-Compassion Scale – short form (SCS-SF)* includes 12 items, assessing a person’s ability to hold their feelings of suffering. It encompasses three uncompassionate and three compassionate approaches, such as showing kindness, rather than being harsh on oneself, recognizing the shared human experience of being imperfect, and focusing on one’s mindful experience in the moment [42]. The items are rated on a 5-point Likert scale ranging from 1 (“almost never”) to 5 (“almost always”). The scale generates a total mean score ranging from 12 to 60, with higher scores indicating higher levels of self-compassion. The SCS-SF is a short version of the original instrument [40]. In a Swedish sample with older adults [12], acceptable internal consistency was reported (Cronbach’s alpha: .68), while the internal consistency was good in the current study (alpha value = .88).

### Analyses

Data were screened for outliers and the data distribution for each variable was inspected. No outliers were identified, and all distributions were normal – except that for the HSCL-25, which had a positive skew. The HSCL-25 data were therefore inverse-transformed (1/x) before analysis, but since this did not affect the results, the original data were used in all analyses.

The factor structure of the SE-DT was investigated through explorative factor analysis using principal component analysis with promax rotation [52]. Associations between variables were assessed with Spearman’s *rho*, internal reliability with Cronbach’s alpha and test-retest with intra-class correlation, using a two-way mixed effects model with absolute agreement. Correlation matrices were calculated with polychoric correlations since the data were ordinal-scaled. Using polychoric correlations estimates latent variables more accurately than their ordinal expressions. Bartlett’s test of sphericity [7] was used to check that the correlation matrix was not random, and a KMO statistic [23] above .50 was required.

Comparisons between groups were made with independent t-tests or Mann-Whitney U-tests, depending on the data structure and measures of effect were calculated with Cohen’s *d,* pooled to correct for differences in sample size*.* A *p* value < .05 was regarded as the significance threshold in all analyses.

## Results

### Factor structure

The exploratory factor analysis suggested a one-factor solution with an eigenvalue of 5.83 that explained 58.28% of the variance. All items had factor loadings of between .70 and .83 on this single factor. It was the only solution with an eigenvalue above 1, so no further factor analyses were conducted.

### Construct validity

The mean score and standard deviation for each instrument are presented in Table 3. The correlations between the SE-DT and the other instruments were significant and in the hypothesized directions (Table 4), i.e., analyses showed a strong positive correlation with general self-efficacy (GSE) and a moderate positive correlation with self-compassion (SCS-SF), whereas there were moderate negative correlations with difficulties with emotion regulation (DERS-16), psychiatric symptoms (HSCL-25), and BPD symptoms (BSL-23).

**Table 3:** Mean scores, standard deviations, and ranges for each instrument used in this study

Variable	Non-clinical (*n* = 407)			Clinical (*n* = 46)		
	*M*	SD	Range	*M*	SD	Range
SE-DT	27.11	5.97	10–40	16.63	4.28	10–28
GSE	28.58	5.51	10–40	n/a	n/a	n/a
SCS-SF	36.55	9.09	12–60	n/a	n/a	n/a
HSCL-25	1.89	0.63	1.00–3.88	n/a	n/a	n/a
DERS-16	36.01	14.14	16–80	61.60	10.62	40–80
BSL-23	0.92	0.78	0.00–3.61	1.51	0.72	0–2.91

*Note*. *SE-DT* Self-Efficacy in Distress Tolerance scale, *GSE* General Self-Efficacy scale, *SCS-SF* Self-Compassion Scale – short form, *HSCL-25* Hopkins Symptom Checklist – short version, *DERS-16* Difficulties in Emotion Regulation Scale – brief version, *BSL-23* Borderline Symptom Checklist – short version

**Table 4:** Correlations (Spearman’s *rho*) between the SE-DT and the other instruments in the non-clinical community sample (*n* = 407)

Variable	*rho*	*p*
GSE	.81	<.001
SCS-SF	.61	<.001
HSCL-25	−.50	<.001
DERS-16	−.58	<.001
BSL-23	−.52	<.001

*Note*. *SE-DT* Self-Efficacy in Distress Tolerance scale, *GSE* General Self-Efficacy scale, *SCS-SF* Self-Compassion Scale – short form, *HSCL-25* Hopkins Symptom Checklist – short version, *DERS-16* Difficulties in Emotion Regulation Scale – brief version, *BSL-23* Borderline Symptom Checklist – short version

### Reliability

The internal reliability of the SE-DT was high (alpha = .92) with inter-item correlations ranging from .40 to .67. All items correlated moderately (*r* = .63–.78) with the scale total and no deletion of items would improve the internal consistency markedly. The test-retest reliability was ICC = .74.

### Difference between the non-clinical and the clinical samples

The mean value of the SE-DT in the CP sample (*M* = 16.63, SD = 4.28) was significantly lower (*t*(432) = 8.97, *p* < .001, *d* = 1.45) than that in the NC sample (*M* = 27.11, SD = 5.97). In the CP sample, the DERS-16 was significantly negatively correlated with the SE-DT (*rho* = −.46, *p =* .02); the BSL-23 also showed a negative correlation, but it was not significant (*rho* = −.27, *p* = .15).

### Sensitivity to change

Participants who completed a brief or a full-scale skills training group (*n* = 27) reported a significant improvement in SE in regard to DT (*t*(19) = 4.89, *p* = .001, *d* = 0.82), when comparing the SE-DT scores before (*M* = 16.63, SD = 4.28) and after (*M* = 22.52, SD = 5.40) treatment. However, there were no statistical improvements in scores for emotion regulation measured with the DERS-16 (*t*(19) = 1.23, *p* = .24, *d* = 0.31) or borderline symptoms measured with the BSL-24 (*t*(19) = 1.37, *p* = .18).

## Discussion

The aim of the present study was to evaluate the psychometric properties of the SE-DT, assessing a person’s perceived ability to handle distress caused by emotional crises. The SE-DT focuses on the use of effective behaviors (skills), as opposed to dysfunctional behaviors with negative consequences (such as self-harm) or which one regrets [31]. It is designed to facilitate the investigation of DT skills’ specific contributions to treatment effect and dropout in, for example, standalone skills training groups and skills training in full-scale DBT programs. Overall, the SE-DT showed adequate to good psychometric properties, displaying a single-factor structure with high internal consistency, good test-retest reliability, and moderate to strong correlations with other psychiatric instruments, in the hypothesized directions. The moderate correlations with some psychiatric instruments indicate that these instruments have a moderate and expected partial overlap with the SE-DT, but that they measure different constructs. The SE-DT therefore displays an adequate divergent validity when comparing instruments that assess variables relevant to psychiatric problems. The strong correlation with the GSE indicates good convergent validity, while the fact that the correlation was not perfect suggests that the GSE cannot completely replace the SE-DT, and that the SE-DT adds important information in investigation of the specific SE in DT. Overall, these results are in line with previous investigations of the original GSE, indicating that it is a reliable, homogeneous, and unidimensional scale [45]. In addition, the SE-DT could identify differences between a NC community sample and a CP sample with BPD patients and patients with borderline features, with lower scores on the SE-DT in the CP sample. This result was expected, since it is known that SE typically shows a negative association with psychiatric problems such as depression, anxiety, and self-harm [6, 36]. The SE-DT also showed an ability to detect improvements in patients’ belief in their ability to tolerate distress after a brief skills training. This result is in accordance with several treatment studies, reporting that skills training can improve BPD patients’ ability to perform skillful behaviors [37], and that higher levels of SE are associated with less frequent self-harm [6]. Interestingly, the SE-DT seemed to be more sensitive to change than other instruments used to evaluate skills, such as the DERS-16 and the SCS-SF. The reason for this could be that it is more complicated or takes longer to learn how to regulate specific emotions or develop self-compassion than to deal with distress and emotional crises, i.e., the SE-DT might be able to detect change at an earlier stage in the treatment process. However, these results must be replicated in a treatment study where a single treatment program is evaluated. In the present study, patients from different treatment interventions, targeting the improvement of emotion regulation and/or DT skills, were included. The results are therefore difficult to interpret with regard to this question.

As expected, the SE-DT (measuring domain-specific SE) showed the strongest association with the GSE (measuring general SE). This does not come as a surprise, since the two scales share similar item formulations. However, a domain-specific instrument like the SE-DT is believed to be a stronger predictor of specific behaviors such as effective DT skills. In addition, the aspects separating the two instruments might prove to be of particular importance in treatment studies where a domain-specific instrument is expected to show higher sensitivity to change than an instrument assessing general SE. Investigating this assumption further would require a study comparing the SE-DT and the GSE in a clinical sample during an evaluation of a treatment intervention.

The positive association between the SE-DT and self-compassion (SCS-SF) was also in line with expectations, since self-compassion involves showing warmth, understanding, and kindness to oneself, in particular in times of difficulties and suffering [40], which could strengthen the belief in one’s own ability to handle difficulties. Indeed, self-compassion has been suggested to protect a person’s SE in face of failures, and results have shown a positive association between the two variables [29].

The negative association between the SE-DT and difficulties in emotion regulation measured with the DERS-16 [8] was only moderate, even though both DT and emotion regulation represent important skills in DBT. However, a person can deal effectively with distress and emotional crises, for example, by distracting themself, without necessarily knowing how to regulate specific emotions. When levels of distress are high, it is difficult to be mindful of the emotion and making a decision on how to regulate it.

The SE-DT was negatively associated with the HSCL-25 (depression and anxiety) and the BSL-23 (symptoms of BPD) in the non-clinical sample. This was unsurprising, given that people with psychiatric problems would be expected to show low ability to handle distress. However, it may be surprising that the correlation between the SE-DT and the BSL-23 was not significant in the clinical sample. It is worth noting that the clinical sample was small and the analysis may have lacked statistical power to detect weak correlations. Another explanation could be that the association is non-linear and therefore levels out when one has reached a certain level of BPD symptoms. Explorative post-hoc analyses of non-linear associations between the variables were all non-significant. Again, the small sample size resulted in low statistical power for these analyses. Further, there may be other variables, such as the ability to regulate emotions (see also above), that are more important in explaining why some patients remain at high levels of borderline-typical problems. This needs to be further investigated in much larger samples.

The adaptation of the scale from the GSE to the SE-DT was made by two of the authors. There was no consultation with any outside specialists in this process, which might be a limitation worth noting. Also, the self-selected community sample and the small CP sample cause difficulties in interpreting and generalizing the results in the current study. There is always a risk of bias in studies with self-selected participants and it is difficult to say how this may have affected the current evaluation. However, the data distributions were, with some exceptions, satisfactory and there were no signs of floor or ceiling effects. Most of the variables, including emotion regulation, are relevant in the normal population and collecting data from NC community samples is therefore adequate. In spite of the small size of the CP sample, the results were significant when comparing groups, as well as pre- and post-treatment assessments. However, the results of this study should be replicated in larger clinical samples. Research investigating both BPD patients and patients with other diagnoses reporting self-harm would add valuable knowledge to this research field. In addition, since the CP sample consisted mostly of female participants, efforts should be made to include male patients. Nevertheless, these limitations are less problematic in a psychometric evaluation than for example in evaluation of treatment efficacy. Lastly, the research field would benefit from a common definition of DT and an analysis of the advantages of using domain-specific SE instruments (compared with general SE instruments) when investigating changes in treatment studies.

## Conclusion

The present study provides preliminary evidence for the usefulness of a scale assessing self-efficacy in distress tolerance, showing it to be a valid and reliable instrument and indicating that the total score should be recommended for use. Future research should focus on evaluating the scale in a larger clinical sample, including male patients and patients with different diagnoses, preferably investigating the scale’s ability to detect change. In particular, the aim should be to investigate distress tolerance skills’ specific contributions to treatment outcome and dropout in standalone skills training and skills training in dialectical behavior therapy.

## Data Availability

Data will not be made publicly available due to confidentiality, but can be made available upon reasonable request to the corresponding author.
